# Minimizing Contact Resistance and Flicker Noise in Micro Graphene Hall Sensors Using Persistent Carbene Modified Gold Electrodes

**DOI:** 10.1021/acsami.4c05451

**Published:** 2024-06-08

**Authors:** Honglin Sun, Ting Huang, Md Masruck Alam, Jingwei Li, Dong Wook Jang, Tianle Wang, Haohan Chen, Yi-Ping Ho, Zhaoli Gao

**Affiliations:** † Department of Biomedical Engineering, The Chinese University of Hong Kong, Shatin, New Territories 999077, Hong Kong SAR, China; ‡ Department of Chemical and Biological Engineering, The Hong Kong University of Science and Technology, Clear Water Bay, Kowloon 999077, Hong Kong SAR, China; § School of Biotechnology, Jiangnan University, 1800 Lihu Avenue, Wuxi 214122, China; ∥ Centre for Novel Biomaterials, The Chinese University of Hong Kong, Shatin, New Territories 999077, Hong Kong SAR, China; ⊥ Hong Kong Branch of CAS Center for Excellence in Animal Evolution and Genetics, The Chinese University of Hong Kong, Shatin, New Territories 999077, Hong Kong SAR, China; # State Key Laboratory of Marine Pollution, City University of Hong Kong, Kowloon Tong, Kowloon 999077, Hong Kong SAR, China; ∇ Shun Hing Institute of Advanced Engineering, The Chinese University of Hong Kong, Shatin, New Territories 999077, Hong Kong SAR, China; ¶ CUHK Shenzhen Research Institute, Nanshan, Shenzhen 518172, China

**Keywords:** Graphene, 2D Materials, Magnetic Sensing, Micro Hall Sensor, Contact Noise

## Abstract

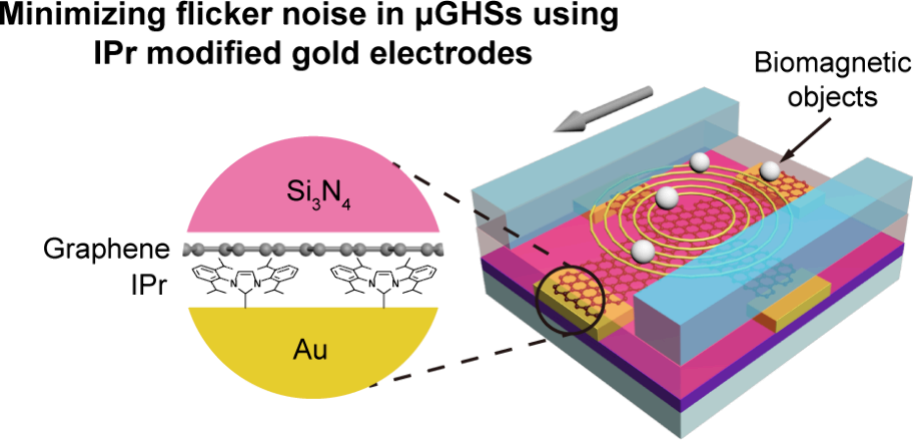

Scalable micro graphene Hall sensors (μGHSs) hold tremendous potential for highly sensitive and label-free biomagnetic sensing in physiological solutions. To enhance the performance of these devices, it is crucial to optimize frequency-dependent flicker noise to reduce the limit of detection (LOD), but it remains a great challenge due to the large contact resistance at the graphene–metal contact. Here we present a surface modification strategy employing persistent carbene on gold electrodes to reduce the contact resistivity by a factor of 25, greatly diminishing μGHS flicker noise by a factor of 1000 to 3.13 × 10^–14^ V^2^/Hz while simultaneously lowering the magnetic LOD *S*_*B*_^1/2^ to 1440 nT/Hz^1/2^ at 1 kHz under a 100 μA bias current. To the best of our knowledge, this represents the lowest *S*_*B*_^1/2^ reported for scalable μGHSs fabricated through wafer-scale photolithography. The reduction in contact noise is attributed to the π–π stacking interaction between the graphene and the benzene rings of persistent carbene, as well as the decrease in the work function of gold as confirmed by Kelvin Probe Force Microscopy. By incorporating a microcoil into the μGHS, we have demonstrated the real-time detection of superparamagnetic nanoparticles (SNPs), achieving a remarkable LOD of ∼528 μg/L. This advancement holds great potential for the label-free detection of magnetic biomarkers, e.g., ferritin, for the early diagnosis of diseases associated with iron overload, such as hereditary hemochromatosis (HHC).

## Introduction

**Figure 1:**
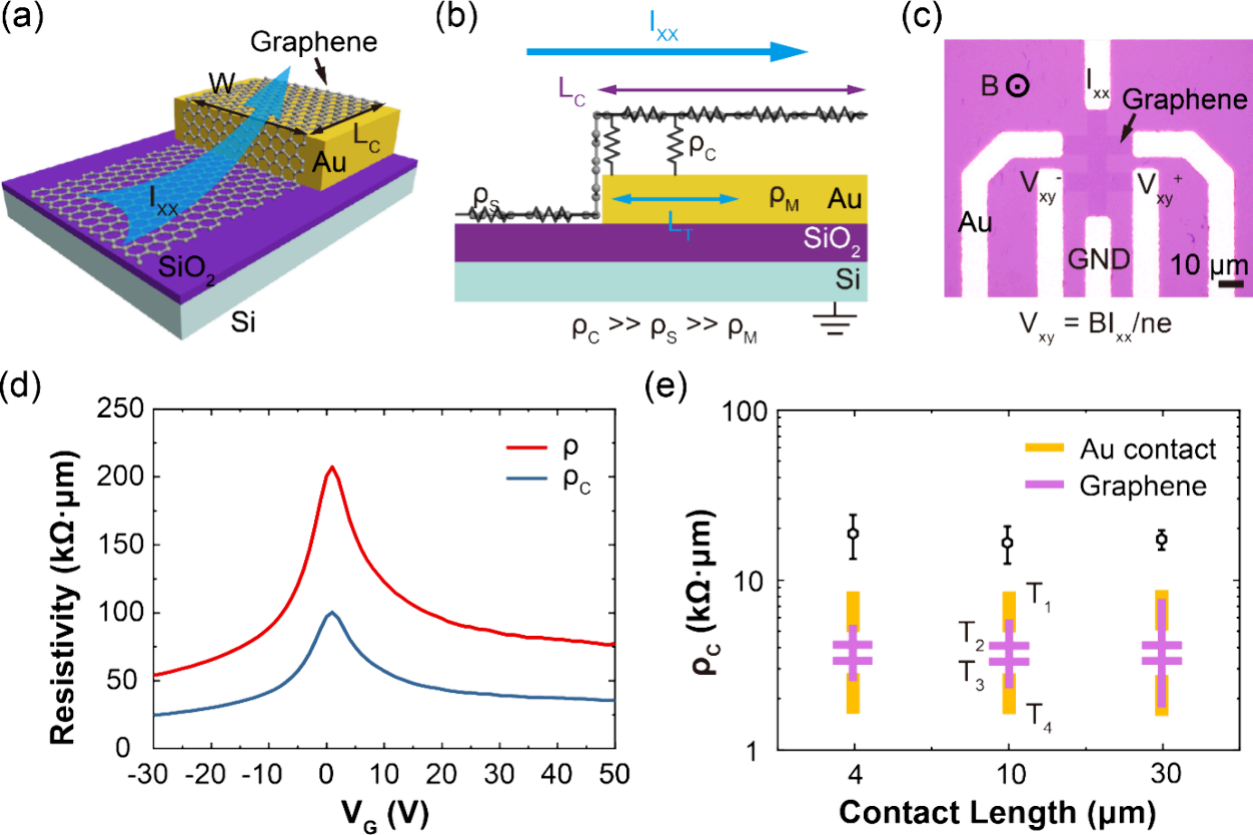
CCE and contact resistance in μGHS fabricated through photolithography. (a) Schematic showing the graphene-gold contact of the micro graphene Hall sensor (μGHS) with a contact width of *W* and a contact length of *L*_*C*_. (b) Current injection from graphene to gold occurs within a limited length *L*_*T*_ for a contact length of *L*_*C*_, leading to the CCE. (c) Optical image of a μGHS device with a six-terminal Hall bar structure, fabricated on a 285 nm SiO_2_ substrate. The GND represents the ground terminal. The formula below is the definition of Hall effect, where *V*_*xy*_ is the Hall voltage, *I*_*xx*_ is the bias current, *B* is the vertical magnetic field, *n* is carrier density, and *e* is elementary charge. (d) Evolution of μGHS contact resistivity against various back gate voltages, peaking near the CNPs. The minimum resistivity, attained at a back gate voltage of −30 V, is recorded at approximately 24.7 kΩ·μm. (e) Contact resistivity (ρ_*C*_) of μGHSs plotted against contact length (*L*_*C*_). T_1_, T_2_, T_3_, and T_4_ indicate four different terminals for the measurement of ρ_*C*_. The ρ_*C*_ remains consistent across different *L*_*C*_, suggesting a CCE within the μGHS.

Magnetic substances present in body fluid, particularly ferritin, hold significant promise as biomarkers for the label-free diagnosis of early stage diseases, such as hereditary hemochromatosis (HHC).^1−6^ A micro Hall detector (μHD) offers a high signal-to-noise ratio (SNR), excellent linearity in magnetic response, and CMOS compatibility, making it a promising platform for biomagnetic sensing.^7−17^ The performance of the μHD is expected to be further enhanced when integrated with two-dimensional (2D) graphene materials. Unlike traditional semiconducting materials, graphene exhibits increased carrier mobility as carrier density decreases,^18−20^ a unique characteristic making it an ideal nanomaterial for scalable fabrication of μHDs with high performance. However, the efforts to reduce the limit of detection (LOD) for micro graphene Hall sensors (μGHSs) face significant challenges primarily due to flicker noise (1/f noise),^21−25^ which arises from two key factors: fluctuations in carrier mobility within the graphene channels,^26^ and current crowding effect (CCE) mediated contact noise at the graphene–metal interface^27^ (Figure 1a, 1b). Both aspects contribute to the flicker noise, complicating the advancement of μGHSs. These complications become particularly pronounced in scalable graphene sensor arrays fabricated through photolithography which exhibit large contact resistance due to mismatches in work function between graphene and metal contacts, as well as their weak van der Waals interactions.^28−31^ To minimize noise signals and contact resistance in scalable μGHSs, it is crucial to optimize the work function discrepancy and enhance the interactions between graphene and metal electrodes.

Here, we report on the application of persistent carbene to mitigate contact noise at the graphene-gold electrode interface, achieving a reduction in the flicker noise of the μGHS by a factor of 1000 to 3.13 × 10^–14^ V^2^/Hz and an improvement in the magnetic LOD *S*_*B*_^1/2^ to 1440 nT/Hz^1/2^ at 1 kHz with a 100 μA bias current. Remarkably, our approach significantly reduces contact resistance by an order of magnitude and decreases flicker noise in μGHSs by a factor of 5 as compared to previous reports.^11,28,32^ These enhancements are ascribed to the π–π stacking interaction and reduced work function difference between the persistent carbene-treated gold electrode and graphene, as verified through Kelvin probe force microscopy (KPFM) analysis. By integrating a microcoil into the μGHS, we have achieved real-time detection of superparamagnetic nanoparticles, marking a significant advancement in detecting magnetic biomarkers such as ferritin. Our work paves the way for the scalable production of high-quality, low-noise μGHSs through photolithography, thereby opening new avenues for their application in label-free biomagnetic sensing for early diagnosis of diseases such as hereditary hemochromatosis (HHC).

## Results and Discussion

Micro graphene Hall sensor (μGHS) arrays (Figure 1c, S1a) were fabricated on 4-in. highly p-doped Si substrates with a 285 nm thick SiO_2_ dielectric layer via a scalable photolithography process (see Experimental Methods for details). Monolayer graphene was synthesized on Cu foils via chemical vapor deposition (CVD). The as-grown CVD graphene was of high quality as assessed by Raman spectroscopy and TEM. As shown in Figure S1b, the Raman spectrum featuring a 2D bandwidth (fwhm) of ∼28.3 cm^–1^, confirms the monolayer characteristic of the CVD graphene. The negligible D peak at ∼1350 cm^–1^ with a D/G ratio <0.01 indicates a low defect level in the graphene channel after photolithography. The TEM selected area electron diffraction (SAED) pattern (Figure S1c) reveals graphene’s distinctive 6-fold symmetry and honeycomb lattice. The high quality of as-fabricated graphene devices is further validated by transport measurements of 20 individual μGHSs (Figure S1d), with a carrier mobility μ of 3423 ± 1045 cm^2^/(V·s).

In graphene devices, metal contacts crucially contribute to the flicker noise,^27,33−35^ which hinders the efforts to minimize the LOD of μGHSs. Previous studies have shown that the contact noise increases exponentially with contact resistance (*R*_*C*_).^27,36^ We employed a gated four-terminal measurement (Figure 1e) to characterize the contact resistivity (ρ_*C*_) between graphene and gold electrodes. With a back gate voltage of −30 V, the determined contact resistivity (ρ_*C*_) for an 8 μm wide contact area is ∼24.7 kΩ·μm (see Figure 1d), which accounts for 91% of the graphene channel’s total resistivity. Here, the total resistivity (ρ) of a two-terminal graphene channel is defined as *ρ = 2ρ*_*C*_*+ ρ*_*S*_, where ρ_*S*_ represents the sheet resistivity of the graphene channel. Moreover, the fundamental resistivity discrepancy between graphene (ρ_*S*_) and gold (ρ_*M*_) results in a charge transfer length (*L*_*T*_) that is significantly shorter than the actual contact length (*L*_*C*_) (Figure 1b). It is because the current injection is restricted to the contact edge, where the graphene sheet resistance *R*_*S*_ contributes less to the resistance of entire current injection pathway. This discrepancy leads to a CCE, which further amplifies the impact of contact resistance on flicker noise in μGHSs.^27^ To investigate this phenomenon, we measured the ρ_*C*_ between graphene and gold electrodes across three sets of μGHSs, each featuring different contact lengths. As shown in Figure 1e, despite the increase in *L*_*C*_ from 4 to 30 μm, we observed that the contact resistance remained largely unchanged, substantiating the existence of CCE with a *L*_*T*_ less than 4 μm. These results highlight the critical need to optimize the graphene-metal interface contacts for the development of scalable and low-noise μGHSs.

**Figure 2:**
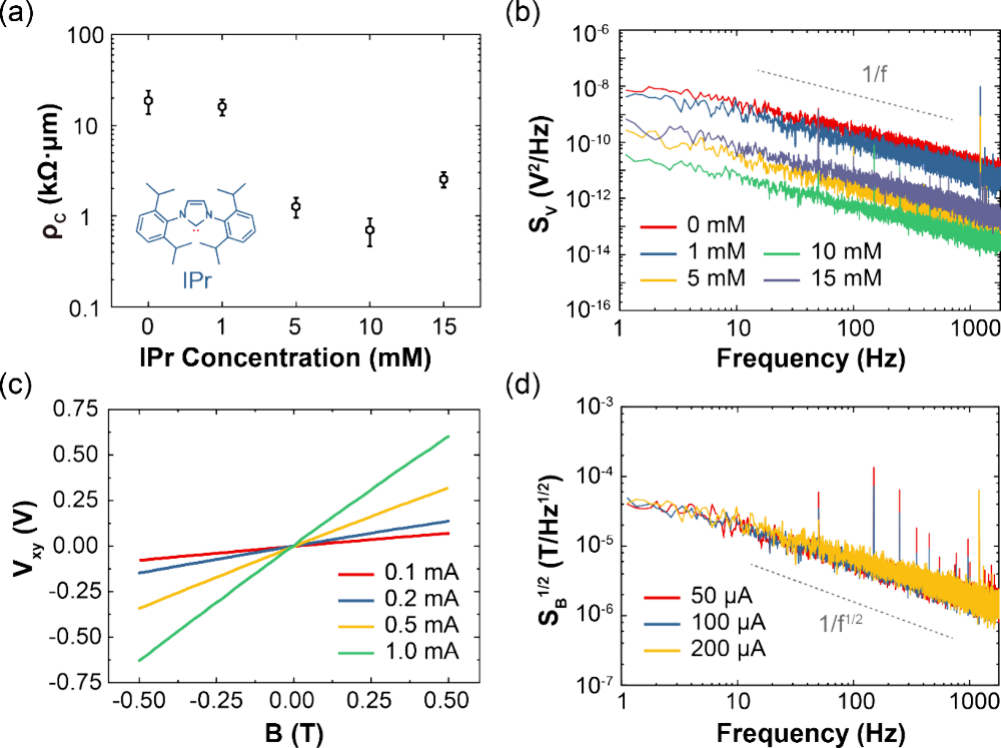
Enhancement of μGHS performance through persistent carbene modification. (a) Contact resistivity of μGHSs at different IPr concentrations. (b) Noise PSD of μGHSs treated with various IPr concentrations, showing a *1*/*f* noise dependence. The noise levels decrease with increasing IPr concentration, showing an optimal value at 10 mM. (c) The Hall response of μGHS, subjected to varying bias currents, is plotted as a function of the external magnetic field, ranging from −0.5 to 0.5 T. (d) The derived magnetic LOD *S*_*B*_^1/2^ of μGHSs exhibit a *1*/*f*^*1/2*^ dependence across the frequency range of 0.2 Hz to 1.83 kHz.

To mitigate flicker noise in μGHS, we employed a persistent carbene, specifically, 1,3-Bis (2,6-diisopropylphenyl) imidazol-2-ylidene (IPr), to functionalize the gold electrode surface and reduce the contact resistance. This stabilized carbene can form a self-assembled monolayer on gold surface,^37−40^ with its bonding strength to gold recorded at 67 kcal·mol^–1^, surpassing that of Au–S bonds.^41^ Previous electronic studies^41,42^ have revealed that the Au-NHC bond provides high conductivity and stability through conjugation, thereby enhancing charge injection efficiency and reducing contact resistance. In our work, we have taken these insights further by demonstrating that modifying gold electrodes with IPr significantly reduces the contact resistivity of the μGHS, and the contact resistivity varies with the concentration of IPr, as shown in Figures 2a and S2. The lowest value was achieved at 10 mM, marking a significant improvement by a factor of 25 over untreated devices, from 18.7 ± 5.4 kΩ·μm (without IPr) to 0.7 ± 0.2 kΩ·μm (with IPr). This represents a remarkable reduction in contact resistivity by an order of magnitude over previous reports.^28^ It is worth noting that, since graphene is transferred onto the gold electrodes after IPr treatment (Figure S3a), the graphene channels remain unmodified by IPr. Compared with existing methods to reduce graphene-metal contact resistivity, such as contact area patterning,^43^ ultraviolet/ozone treatment,^44^ Nickel-etched contact^45^ and van der Waals contact engineering,^28^ our persistent carbene modification approach avoids additional microfabrication processes and is cost-effective while providing low contact resistivity. This result demonstrates the effectiveness of IPr treatment in enhancing the graphene-gold interface and reducing their contact resistance, which is expected to lead to improved flicker noise performance in μGHSs.

To evaluate the effectiveness of IPr treatment in improving the noise performance of μGHS, flicker noise measurements were performed on IPr treated μGHS samples using a lock-in amplifier. Figure 2b shows the noise power spectral density (PSD) *S*_*V*_ spanning a frequency range from ∼0.2 Hz to ∼1.83 kHz for μGHS subjected to varying concentrations of IPr. The *S*_*V*_ exhibits a distinct 1/*f* behavior and increases with *I*_*xx*_ (Figure S3b), in agreement with Hooge’s empirical model.^21,26^ Within the 0–10 mM IPr concentration range, an increase in IPr concentration leads to decreased flicker noise, while further increasing to 15 mM results in an elevated noise level, aligning with the trend of contact resistivity across varying IPr concentrations. For μGHS treated with an optimal IPr concentration of 10 mM, the flicker noise PSD at a 100 μA *I*_*xx*_ bias is found to be 3.13 × 10^–14^ V^2^/Hz, marking a significant enhancement by a factor of 1000 over untreated devices, and by a factor of 5 compared to the scalable μGHSs of previous studies.^11,32^ Our work is the first attempt ever reported to optimize the contact noise of scalable μGHSs, providing a new pathway toward low-noise magnetic field sensing.

The current-related sensitivity, defined as *S*_*I*_*= ∂V*_*xy*_*/(I*_*xx*_*·∂B)*, is a critical metric for assessing the sensitivity of μGHS’s to an external magnetic field *B*. Here, *S*_*I*_ is normalized by the bias current *I*_*xx*_, and *V*_*xy*_, the Hall voltage (Figure 1c), is defined using the equation *V*_*xy*_*= BI*_*xx*_*/(ne)*, where *n* denotes the two-dimensional charge carrier density, *e* is the elementary charge. At room temperatures, we measured *S*_*I*_ of the μGHS under four different bias currents (*I*_*xx*_ = 0.1, 0.2, 0.5, and 1.0 mA), by applying a perpendicular magnetic field ranging from −0.5 to 0.5 T. As illustrated in Figure 2c, the μGHS exhibited an exceptional linear Hall response, with an *S*_*I*_ ∼ 1230 V/(A·T), consistent with the previous report.^46^ This result highlights the effectiveness of our IPr treatment in preserving the *S*_*I*_ of μGHS, due to the negligible change in carrier density for graphene on IPr-treated gold compared to the average carrier density across the entire graphene channel.

The limit of detection (LOD) for the Hall detector, determined by the flicker noise and current-related sensitivity, is expressed as *S*_*B*_^1/2^ = *S*_*V*_^1/2^/(*S*_*I*_*·I*_*xx*_*)*, where *S*_*V*_ represents the noise power spectral density (PSD). As illustrated in Figure 2d, the *S*_*B*_^1/2^ exhibited no dependency on *I*_*xx*_, indicating the LOD remains consistent across varying levels of bias current. The fluctuation of *S*_*B*_^*1/2*^ increases with higher frequency is due to the FFT process and there are more data points at higher frequency, making the fluctuation of *S*_*B*_^*1/2*^ more obvious. The determined *S*_*B*_^1/2^ of ∼1440 nT/Hz^1/2^ sets a new benchmark for the lowest LOD achieved in scalable μGHSs manufactured using photolithography, surpassing previous reports in the literature,^11,12,23^ highlighting the potential for broader application and scalability of our μGHSs.

**Figure 3:**
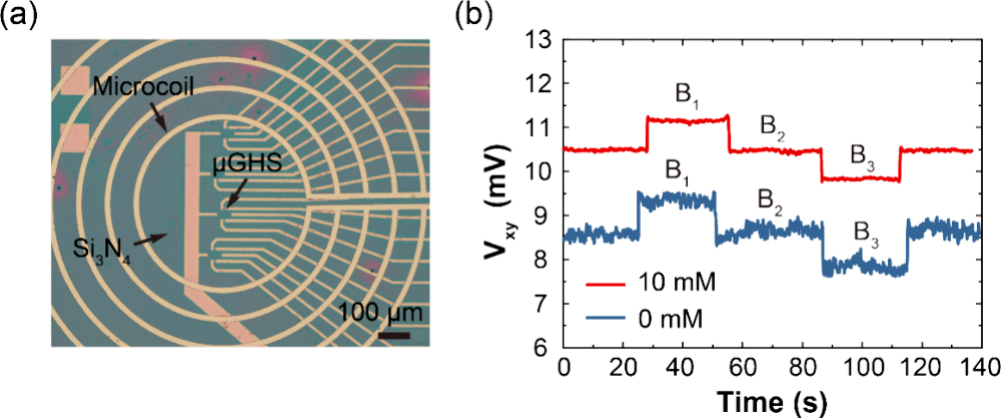
Measurement of SNR in μGHS using a microcoil. (a) Optical image showing a μGHS integrated with a gold microcoil. (b) Comparison of Hall voltage signal fluctuations in μGHS with and without IPr treatment, measured in the time domain under identical magnetic field steps *B*_1_, *B*_2_, and *B*_3_, provided by driving different currents (*I*_*coil*_) through the microcoil.

To further demonstrate the benefits of our IPr treatment in practical magnetic sensing, we compared the Hall voltage fluctuations of μGHSs with and without IPr treatment across different magnetic fields (*B*_1_ ∼ 165 μT, *B*_2_ ∼ 132 μT and *B*_3_ ∼ 99 μT, referring to Figure S4a) generated by the gold microcoil (Figure 3a), which was integrated with the μGHS through a one-step photolithography process (see Supplementary Note 1 for details). As shown in Figure 3b, the Hall voltage signals accurately reflect the change in strength in magnetic fields. Moreover, the Si_3_N_4_ passivation process impacts the contact resistivity and p-dopes graphene channel (Figure S6). The μGHSs treated with IPr exhibited a significant decrease in noise amplitude across varying magnetic fields. The quantified SNR of μGHS treated with the optimal 10 mM IPr is 24.6 dB, notably higher than the 11.1 dB SNR of the untreated devices, highlighting the enhanced magnetic field sensing capability of IPr-treated μGHSs.

**Figure 4:**
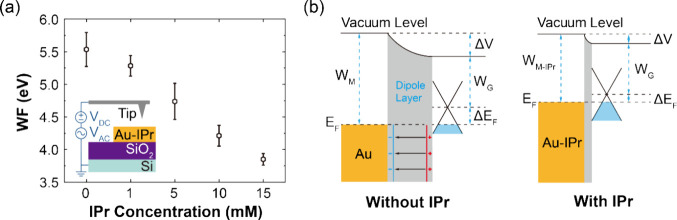
Reduction mechanism of contact resistivity and flicker noise in IPr treated μGHS. (a) WF of gold electrode plotted against IPr concentrations, showing a decrease in WF with increasing IPr treatment concentrations. Inset shows the experimental setup of KPFM. (b) Band structure diagrams comparing vdW contact (without IPr, left) and π–π stacking contact (with IPr, right), illustrating how IPr treatment reduces tunnel barrier of the dipole layer. *W*_*M*_, *W*_*G*_, *W*_*M-IPr*_ represents the WF of gold, graphene, and IPr-modified gold, respectively. *ΔV* is the potential change of the vacuum level bending, *E*_*F*_ is the Fermi level, and *ΔE*_*F*_ is the shift of graphene Fermi level.

To investigate the mechanism of IPr treatment on noise reduction, we employed KPFM to assess the work function (WF) changes in gold electrodes before and after IPr exposure, subjected to different concentrations of IPr. As shown in Figure 4a, the measured WF of the untreated gold electrodes is 5.5 ± 0.3 eV. After IPr treatment, a notable reduction in WF was observed, with the value of 4.2 ± 0.2 eV for 10 mM IPr, comparable to the WF of 4.26 ± 0.02 eV of the CVD monolayer graphene used in μGHSs (Figure S3c). This result reveals a distinct relationship between the WF of gold and its surface functionalization with IPr. As illustrated in Figure 4b, when graphene comes into contact with gold, the mismatch in work function causes electrons transfer from graphene to gold, resulting in a p-type doping effect on the graphene. This interaction leads to a shift of the Fermi level (*ΔE*_*F*_) and the formation of a dipole layer, which impedes further electrons transfer at the graphene-gold interface.^29^ The IPr functionalization modulates the WF of gold to more closely align with that of graphene, thereby reducing the tunnel barrier associated with the dipole layer. Moreover, in μGHSs fabricated via photolithography, where CVD graphene is transferred onto prefabricated gold electrodes, the weak vdW interaction between the graphene and the gold results in a low probability of carrier tunneling.^28^ IPr treatment can enhance the tunneling efficiency at the graphene-gold interface through π–π stacking interaction between the graphene and the IPr, thereby reducing contact resistance and flicker noise in μGHSs. Additionally, this mechanism explains the increase of ρ_*C*_ with 15 mM IPr treatment in Figure 2a. Excess IPr treatment, i.e. Fifteen mM, further reduced the WF of gold to 3.8 ± 0.1 eV, which led to an increased mismatch with the WF of graphene 4.26 ± 0.02 eV and a degraded ρ_*C*_.

**Figure 5:**
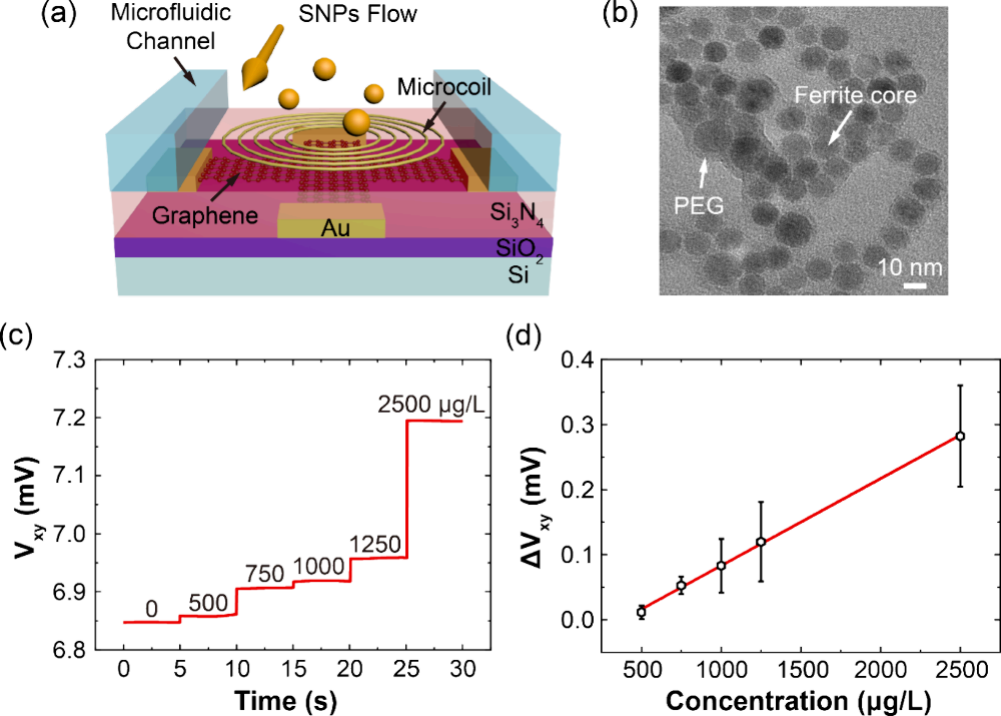
Real-time detection of SNP dispersions using μGHSs. (a) Schematic showing the components and structure of a μGHS integrated with a microcoil and microfluidic channel. (b) TEM bright field image of SNPs, showing ferrite core of SNP with an average diameter of 10 nm, coated by a PEG layer. (c) Real-time Hall voltage signal trace of SNPs dispersions at various concentrations. The insert schematic showing the integration of a microcoil and microfluidic channel into the μGHS setup. (d) Hall signal versus SNP concentrations showing a linear relationship after background subtraction of DI water response.

The development of scalable μGHS with reduced contact resistance and flicker noise marks a significant advancement in the field of biomagnetic sensing. Ferritin, an iron-storage protein (∼474 kDa) comprising of a protein shell and a superparamagnetic ferrite core,^4,47,48^ emerges as a promising biomarker for early diagnosis of HHC in a label-free and minimally invasive setting.^1−6^ In our work, we employed dispersions of PEG-coated superparamagnetic nanoparticles (SNPs) with an average diameter of 10 nm to mimic ferritin (Figure 5b). This approach allowed us to readily evaluate the biomagnetic sensing performance of μGHS. To enable the real-time detection of SNPs, we integrated a polydimethylsiloxane (PDMS) microfluidic channel, fabricated using standard soft lithography techniques,^49^ with the passivated μGHS via oxygen plasma bonding (Figure 5a, Supplementary Note 2). Five aqueous dispersions of SNPs at varying concentrations were introduced into the as-prepared microfluidic channel to assess the detection capabilities of the μGHS. The Hall voltage signals were processed by a lock-in amplifier. As shown in Figure 5c, the μGHS successfully distinguish between different concentrations of SNPs, as evidenced by the distinct steps in potential observed. The Hall voltage signals of μGHS exhibited a strong linearity (R^2^ = 0.99) and the LOD for SNP dispersions was determined to be ∼528 μg/L (3-sigma rule), as shown in Figure 5d. Previous research highlights that serum ferritin levels above 1000 μg/L indicate a high risk of cirrhosis, the main clinical manifestation of HHC.^1,2,6^ Although the magnetization of biogenic ferritin is typically weaker than that of SNPs,^4,48^ which complicates the direct comparison between their LODs, our μGHS has demonstrated great promise for practical applications in the label-free diagnosis of early stage HHC, suggesting its potential for future clinical use.

## Conclusion

We developed scalable μGHSs with persistent carbene modified gold electrodes to minimize contact resistance and flicker noise. The surface modification of gold electrode with IPr led to a remarkable reduction in contact resistivity by a factor of 25 and a decrease in flicker noise by a factor of 1000. This resulted in an exceptional magnetic LOD of 1440 nT/Hz^1/2^ at 1 kHz with a 100 μA bias current, marking the lowest *S*_*B*_^1/2^ reported for scalable μGHSs fabricated through wafer-scale photolithography. These advancements are attributed to the π–π stacking interaction between graphene and IPr, along with the reduction in the WF of gold induced by IPr treatment, as confirmed by KPFM analysis. By integrating the μGHS with a microcoil and microfluidic channel, we demonstrated real-time detection of SNPs, with a superior LOD of ∼528 μg/L. Our approach paves the way for the label-free detection of magnetic biomarkers such as serum ferritin, which is crucial for the early diagnosis of diseases such as HHC. This low-noise and scalable μGHS is expected to advance the development of miniaturized Hall detectors, opening new avenues for applications in medical diagnostics and beyond.

## Experimental Methods

### μGHS Fabrication

UV photolithography was used to define both electrode patterns and graphene Hall bars. The highly p-doped 4-in. Si wafer with a 285 nm thermal oxide layer was immersed in the 1:1 HMDS/acetone solution for 15 h. After hydrophobic modification, the wafer was rinsed with acetone and IPA, then dried with N_2_ flow. The SiO_2_/Si wafer was baked at 115 °C for 2 min, before the photolithography process, to remove unbound HMDS. Electrodes (45 nm Au/8 nm Cr) were fabricated by a lift-off process. A monolayer graphene film, synthesized by our previously reported CVD method^50^ on Cu foil (Alfa Aesar, #13382), was “bubbling” transferred onto the SiO_2_/Si substrate with prepatterned electrodes. The chips with the as-transferred graphene film were dried in a fume hood for 6 h, then immersed in acetone overnight to remove PMMA residues. The chips were spin coated with a photoresist bilayer of PMGI (MicroChem Corp.) and S1813 (Shipley) to improve device fabrication quality.^51^ Typical six-terminal Hall bars (8 μm wide) were defined by another photolithography process. Subsequently, the uncovered graphene area was etched by oxygen plasma (100 sccm, 50 W, 35s). The photoresist residues on the graphene channel were removed by NANO Remover PG (MicroChem Corp.). Finally, the μGHSs were vacuum annealed with 200 sccm Ar and 50 sccm H_2_ at 200 °C for 1 h.

### Contact Resistivity Measurement

The gated four-terminal measurement (Keithley 4200A) was conducted to measure the contact resistivity ρ_*C*_ of μGHSs. The gate voltage *V*_*G*_ was first swept from −30 to 50 V with a 0.1 V bias voltage *V*_*xx*_ between T_1_ and T_4_ to obtain the typical two-terminal *I*_*DS*_*/V*_*G*_ curve of μGHS, and the total resistivity ρ was calculated from this curve. To calculate the graphene channel resistivity ρ_*S*_, we applied a 10 μA bias current between T_1_ and T_4_ while recording the gated voltage change between T_2_ and T_3_. According to the dimension of μGHS, the graphene channel resistivity between T_2_ and T_3_ is one-third of the ρ_*S*_, and the ρ_*C*_ was derived from ρ_*C*_*= (ρ - ρ*_*S*_*)/2*.

### IPr Treatment

By dissolving different amounts of IPr powder (Sigma-Aldrich 696196) in 10 mL of toluene, we prepared six different concentrations (1 mM, 3 mM, 5 mM, 10 mM, 15 mM) of IPr solutions. We submerged prepatterned μGHS gold electrodes in IPr solutions for 24 h. After the reaction, chips were rinsed with THF, acetone and IPA for 1 min each, then dried with N_2_ flow. This washing process is efficient confirmed by AFM characterization and there is no obvious aggregation of IPr residues on the SiO_2_ surface (Figure S5). The fabrication process of IPr-treated μGHS is the same as the graphene transfer and photolithography methods mentioned above.

### Noise Measurement and SNR Evaluation

For noise measurement, the μGHS was biased by DC currents (Keithley 6221) and differential signals from the output electrodes were analyzed by a lock-in amplifier (Zurich Instruments MFLI). The built-in preamplifier is in AC coupling mode, providing larger gain for noise measurements. We recorded *V*_*xy*_ without *B* at 3.66 kHz with 16384 points per trace in the time domain, and the Fast Fourier Transform (FFT) of each time trace was computed with a Hann window. Various *V*_*xy*_, biased by different *I*_*xx*_ ranging from 1 μA to 100 μA, were recorded in 10 time traces respectively and the frequency domain signals were overlapped to reduce variance. For the SNR evaluation, *I*_*xx*_ was fixed at 100 μA (Keithley 2400), while *I*_*coil*_ alternated within 100, 80, and 60 mA (Keithley 6221) to provide magnetic field steps *B*_1_, *B*_2_ and *B*_3_. The Hall signal traces in the time domain against different magnetic fields were collected by the lock-in amplifier. The magnification of the preamplifier was 1000 in these two measurements.

### Work Function Measurement

The KPFM test was conducted on Multimode 8 (Bruker) in air. The KPFM tip (PPP-EFM (Nanosensors), 75 kHz) was first calibrated by measuring newly exfoliated HOPG, whose theoretical work function (WF) value was 4.5 eV. For KPFM in air, the potential difference *ΔV*_*sp*_ between the tip and sample surface was defined by *eΔV*_*sp*_*= W*_*s*_*– W*_*p*_, where *e* is the elementary charge, *W*_*s*_ and *W*_*p*_ are the work functions of the sample and the tip, respectively.

### Real-Time Detection of SNPs Aqueous Dispersions

PEG (MW 2000) coated SNPs (Aladdin P301814) were diluted with deionized water to create five dispersions of different concentrations of 500, 750, 1000, 1250, 2500 μg/L. These dispersions were injected into the microfluidic channel using a syringe pump (KD Scientific 788101) and subsequently detected by the μGHS. The Hall signals were collected by a lock-in amplifier and the magnification of the preamplifier was 1000. The μGHS was biased by a 100 μA DC current *I*_*xx*_ (Keithley 2400), and the microcoil was biased by a 100 mA 1 kHz *I*_*coil*_ (Keithley 6221). The reference frequency generated by the lock-in amplifier was also 1 kHz, consistent with *I*_*coil*_. A three order low-pass filter with 0.1 Hz bandwidth was applied after signal mixing to extract target signal. We sampled time trace signals at 104.6 Hz for ∼5 s for each SNP concentration. The LOD of SNP dispersions was calculated based on LOD = 3σ/*N*, where σ is the standard deviation of the signal response of DI water and *N* is the slope of the working curve.

## Supporting Information Available

Fabrication of the μGHS arrays, graphene channel characterization, gate tunable contact resistivity details, further discussion of the effects of IPr on graphene and the bias current dependence of flicker noise, detailed discussion about the microcoil, fabrication of the microfluidic channel, AFM images of SiO_2_/Si substrates, impacts of Si_3_N_4_ passivation layer; fabrication and characterization of μGHSs; curves of gate tunable contact resistivity; further discussion of the effects of IPr on graphene and the bias current dependence of flicker noise; detailed discussion about microcoil; fabrication details of the microfluidic channel; finite element analysis results and microcoil calibration results; AFM images of SiO_2_/Si substrates without and with IPr treatment; impacts of Si_3_N_4_ passivation layer on μGHS performance (PDF)
