# Supplementary material for: Minimizing Contact Resistance and Flicker Noise in Micro Graphene Hall Sensors Using Persistent Carbene Modified Gold Electrodes
Source: ACS Appl Mater Interfaces. 2024 Jun 8;16(24):31473–9. doi: 10.1021/acsami.4c05451 (PMC11194764; doi:10.1021/acsami.4c05451)
Supplement: Supplementary file 1 — am4c05451_si_001.pdf [file am4c05451_si_001.pdf]

# Supplementary Information

# Minimizing Contact Resistance and Flicker Noise in Micro Graphene Hall Sensors Using Persistent Carbene Modified Gold Electrodes

*Honglin Sun,[1] Ting Huang,[1] Md Masruck Alam,[1] Jingwei Li,[1,2] Dong Wook Jang,[1] Tianle Wang,[1] Haohan Chen,[1,3] Yi-Ping Ho,[1,4,5,6] and Zhaoli Gao[1,7,8,*]*

[1] Department of Biomedical Engineering, The Chinese University of Hong Kong, Shatin, New Territories, Hong Kong SAR, 999077, China

[2] Department of Chemical and Biological Engineering, The Hong Kong University of Science and Technology, Clear Water Bay, Kowloon, Hong Kong SAR, 999077, China

[3] School of Biotechnology, Jiangnan University, 1800 Lihu Avenue, Wuxi, 214122, China

[4] Centre for Novel Biomaterials, The Chinese University of Hong Kong, Shatin, New Territories, Hong Kong SAR, 999077, China

[5] Hong Kong Branch of CAS Center for Excellence in Animal Evolution and Genetics, The Chinese University of Hong Kong, Shatin, New Territories, Hong Kong SAR, 999077, China

[6] State Key Laboratory of Marine Pollution, City University of Hong Kong, Kowloon Tong, Kowloon, Hong Kong SAR, 999077, China

[7] Shun Hing Institute of Advanced Engineering, The Chinese University of Hong Kong, Shatin, New Territories, Hong Kong SAR, 999077, China

[8] CUHK Shenzhen Research Institute, Nanshan, Shenzhen, 518172, China

* Email: zlgao@cuhk.edu.hk

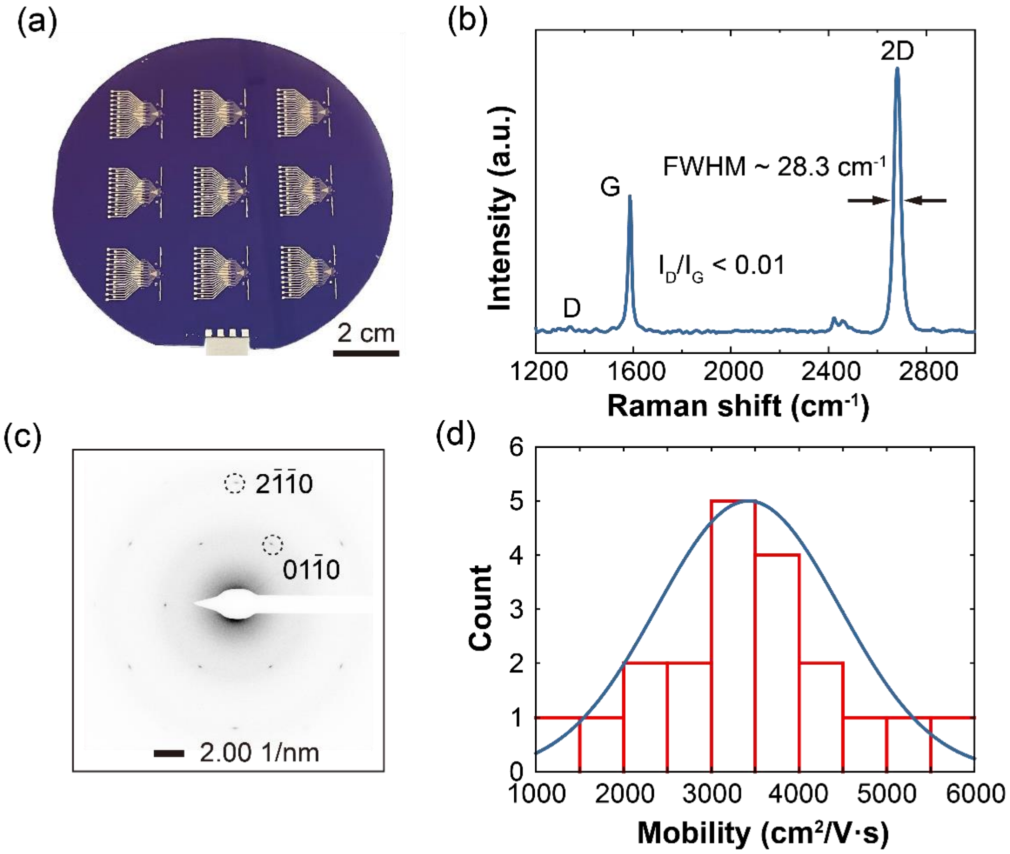


**Figure S1.** Fabrication and characterization of μGHSs. (a) Image of wafer-scale μGHSs arrays on a 4-inch wafer. (b) Raman spectra of graphene channel before and after the photolithographic process. (c) SAED of as-grown CVD monolayer graphene. Typical six-fold symmetry can be observed. (d) Histograms of carrier mobility with Gaussian fits (blue curve) of μGHSs based on 20 individual devices. The average carrier mobility $\mu$ is 3423 ± 1045 $cm^2/(V \cdot s)$.

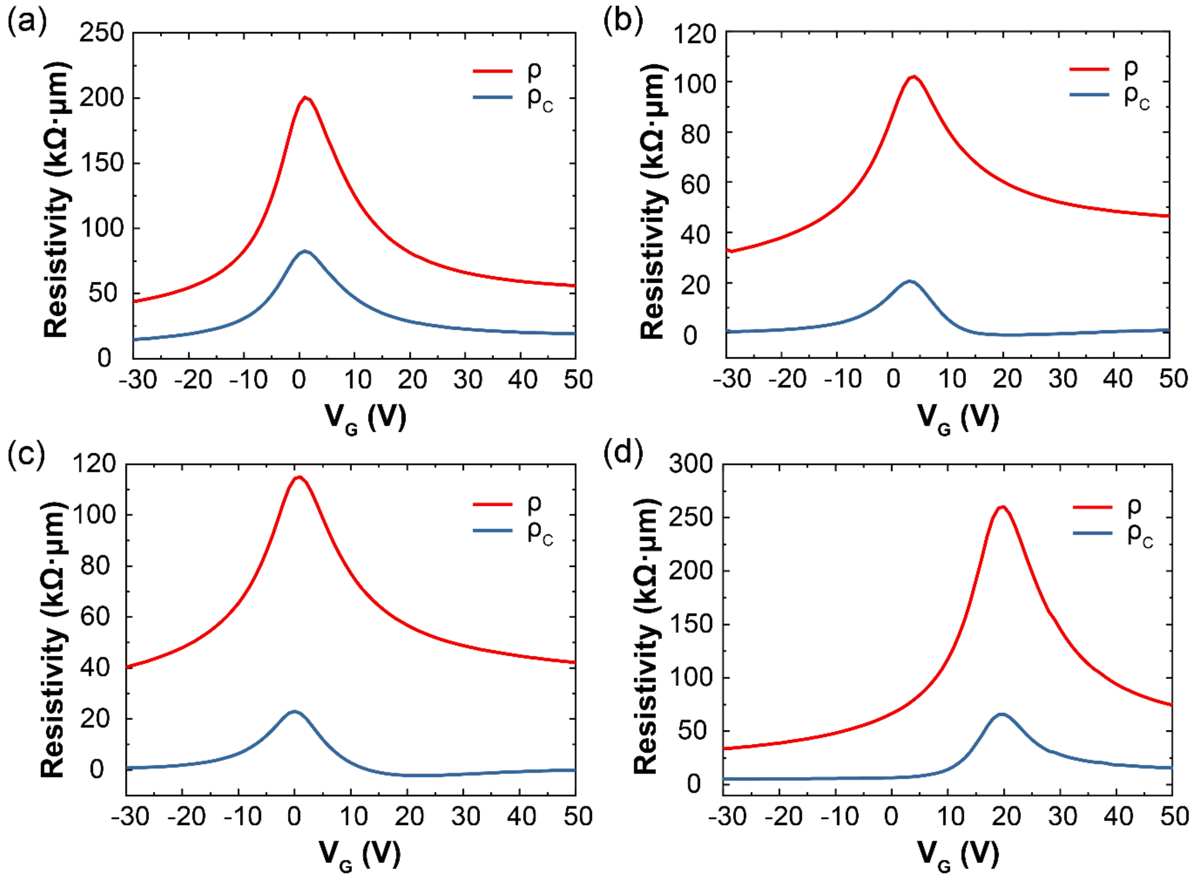


**Figure S2.** Contact resistivity $\rho_C$ of µGHSs varies with back gate voltage and peaks near CNP. IPr concentrations used in (a), (b), (c) and (d) are 1, 5, 10, 15 mM respectively.

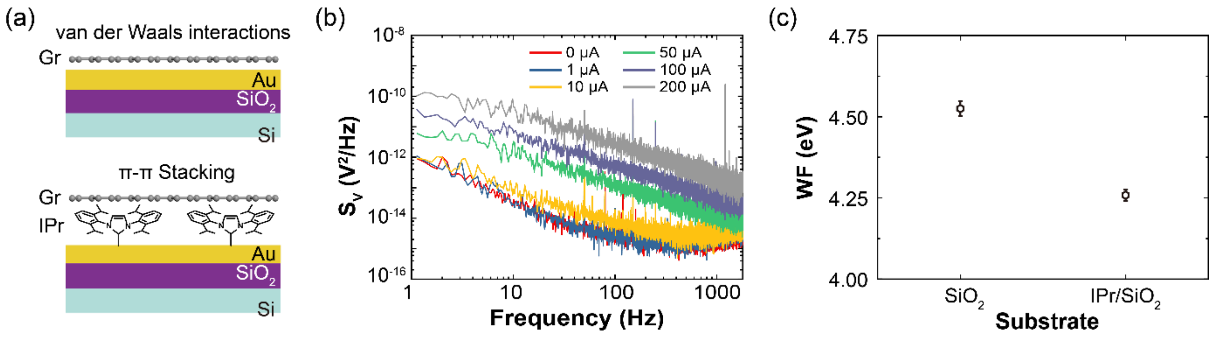


**Figure S3**. (a) The schematic showing the graphene-gold contact interface before and after the IPr modification. (b) The bias current dependence of noise PSD of µGHS treated with 10 mM IPr. When the bias current is less than 10µA, the noise PSD of µGHS (blue and yellow curves)

is limited by the input noise of lock-in amplifier (red curve). (c) The work function (WF) of graphene on pure $SiO_2$/Si substrate and the substrate after 10 mM IPr treatment.

## SUPPLEMENTARY NOTES

### Supplementary Note 1: The fabrication, simulation and calibration of microcoil

Since SNPs inherently lack magnetism in the absence of an external magnetic field, it is necessary to apply a magnetic field for the real-time detection.[1-3] To facilitate device miniaturization and provide a tunable magnetic field, we replaced the bulky electromagnetic coil with a planar microcoil on the μGHS chips (Figure 3a). Before fabricating the microcoil, the μGHS was first passivated by depositing a 100 nm $Si_3N_4$ insulating layer, using PECVD. Before deposition, we first spin-coated a layer of hydrogen silsesquioxane (HSQ) on a graphene device and baked at 80 °C for 4 min to provide a nucleation site for $Si_3N_4$ and to reduce the plasma damage to graphene during the PECVD process. $SiH_4$, $NH_3$ and $N_2$ were used to deposit $Si_3N_4$ film at 250 °C. The gold microcoil, featuring five concentric circles with diameters ranging from 300 μm to 700 μm, was fabricated on the $Si_3N_4$ layer through the lift-off process.

The AC/DC module of COMSOL MULTIPHYSICS (version 5.6) was used to conduct finite element analysis (FEA) of the magnetic field distribution of the microcoil. The material of the microcoil was 8 nm Cr + 45 nm Au. A cubic air domain, with a side length of 3000 μm, was established to simulate the magnetic field distribution in the air, and the outermost 1/10 of it was set to "infinite element domain" to simulate the size of infinite space. The outer surface of the air domain was set to the magnetic insulation boundary. The magnetic field distribution

of the microcoils was simulated with different designs under the same excitation current of 100 mA 1 kHz. The FEA results of our microcoil revealed a centrally symmetric magnetic field distribution and the central magnetic field ($B_{coil}$) is ~165 μT (Figure S4a). Compared to a single-coil structure[4] (Figure S4b), our concentric-coil design promotes a more uniform magnetic field distribution across the plane within the coil. We further calibrated the microcoil by performing lock-in detection of the Hall voltage at various $I_{xx}$. The Hall response signal was collected by a lock-in amplifier (Zurich Instruments MFLI) and the magnification of the preamplifier was 1000. To eliminate the interference of the spurious signal generated by the induced potential, DC $I_{xx}$ was swept (20, 40, 60, 80, 100 μA) instead of AC $I_{coil}$. The microcoil was biased by a 100 mA current $I_{coil}$ (Keithley 6221) during the calibration. As shown in Figure S4c, the results exhibit a robust linear Hall response from the μGHS ($R^2$ = 0.99). The $S_I$ of μGHS was derived through the slope $k = mS_IB_{coil}$, where $m$ represents the magnification of the preamplifier. The derived $S_{I,\ derived}$ = 252 V/AT at 165 μT is close to the $S_I$ = 281 V/AT (Figure S4d) measured by PPMS (Quantum Design, Physical Property Measurement System), yielding a relative error of ~ 10%. This result underscores the reliability of our microcoil and magnetic detection setup.

**Supplementary Note 2: Fabrication of the microfluidic channel**

The intended patterns containing a flow channel were designed by CAD software (AutoCAD 2022), printed on a photomask and transferred onto a silicon wafer spin coated with a 50 μm layer of SU-8 3050 negative photoresists (Kayaku Advanced Materials, Massachusetts, USA). The exposure, development, and baking of the SU-8 mold were conducted according to the manufacturer’s instructions. Polydimethylsiloxane (PDMS) and curing agent (Sylgard 184,

Dow Corning, Michigan, USA) were mixed at a 10:1 (W/W) ratio and cast on the SU-8 mold, following by overnight curing at 55°C. Cured PDMS was detached from the wafer and cut into individual slabs. Inlet and outlet ports were punched using a 1 mm biopsy puncher. The PDMS slabs were cleaned by ethanol followed by water rinsing. The surfaces of passivated μGHS and cleaned PDMS slab were then purged by nitrogen and oxygen plasma treated (Harrick Plasma, New York, USA) for 45 seconds. The two pieces were aligned and bonded under the microscope immediate after the plasma treatment. The bonded assembly was incubated on a hotplate for 10 min at 95°C to strengthen the bonding.

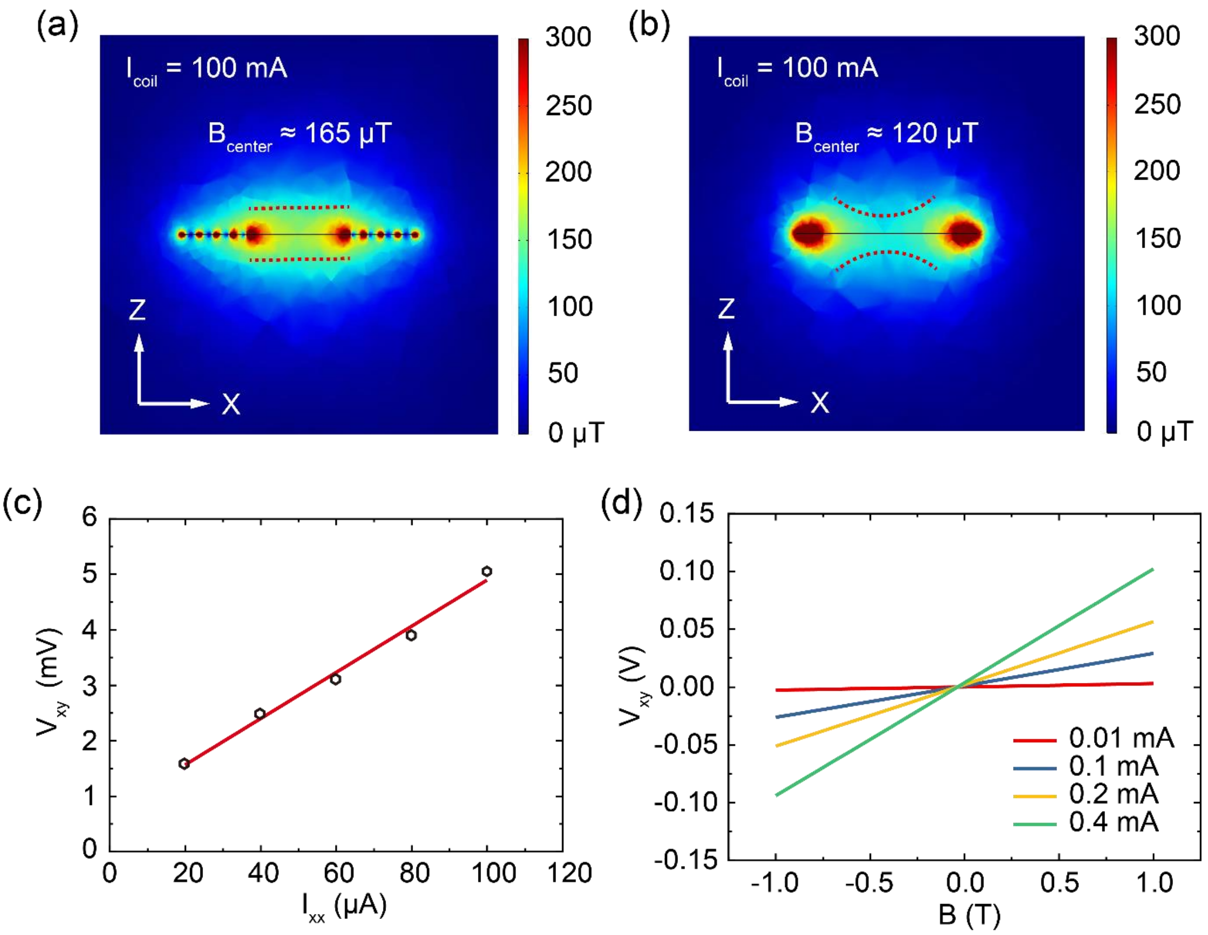


**Figure S4.** Finite element analysis (FEA) and calibration of the microcoil. The FEA results of (a) concentric and (b) single microcoil showing the magnetic field distribution when the

microcoil was biased by a 100 mA current at 1 kHz. The cross-sectional view reveals a centrally symmetric magnetic field distribution. The red dashed lines indicate the 125 μT magnetic field boundary. Both magnetic field distribution uniformity and central strength of concentric microcoil exceed single microcoil. (c) Hall response as a function of bias current under an external magnetic field generated by microcoil. (d) Hall response of passivated μGHS, biased by varying currents, plotted against an external magnetic field ranging from -1.0 T to 1.0 T.

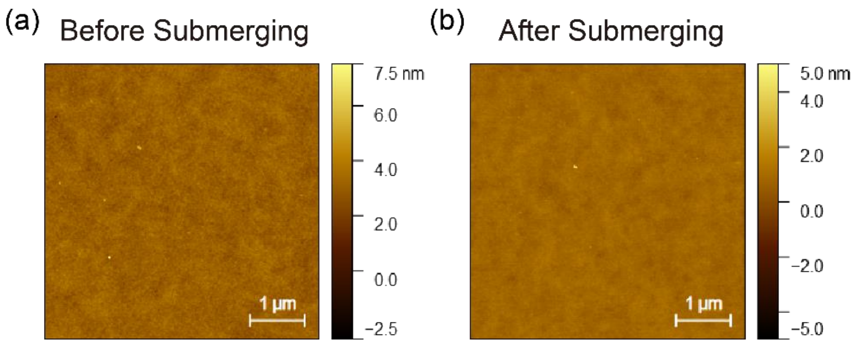


**Figure S5.** The AFM images of the $SiO_2$/Si substrate (a) before and (b) after being submerged in 10 mM IPr solutions for 24 hours.

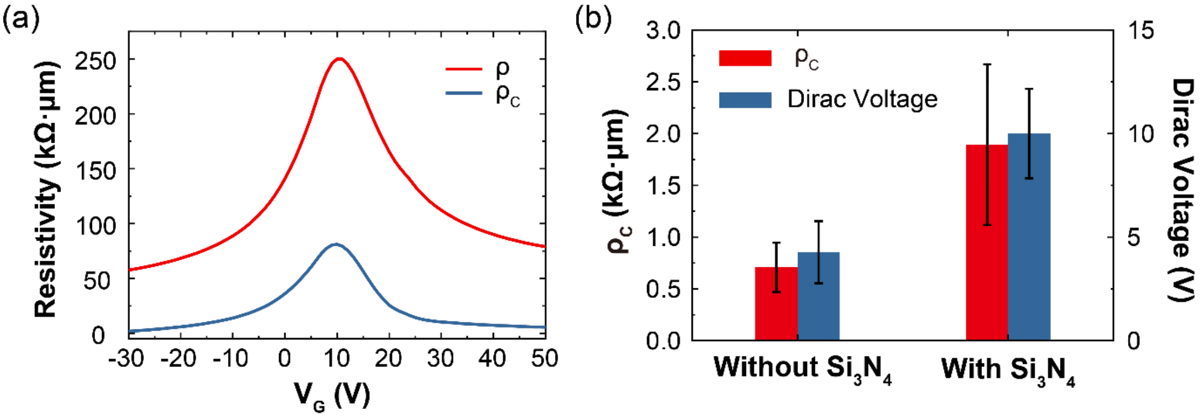


**Figure S6.** The impacts of $Si_3N_4$ passivation layer on the graphene-gold contact and graphene channel. (a) Contact resistivity $\rho_C$ of $Si_3N_4$ passivated μGHSs varies with back gate voltage and peaks near CNP. (b) Comparison of the contact resistivity $\rho_C$ and Dirac voltage of μGHSs

treated with 10 mM IPr before and after $Si_3N_4$ deposition.

## SUPPLEMENTARY REFERENCES

(1) Lee, W.; Joo, S.; Kim, S. U.; Rhie, K.; Hong, J.; Shin, K.-H.; Kim, K. H. Magnetic Bead Counter Using a Micro-Hall Sensor for Biological Applications. *Appl. Phys. Lett.* **2009**, *94* (15), 153903.

(2) Besse, P.-A.; Boero, G.; Demierre, M.; Pott, V.; Popovic, R. Detection of a Single Magnetic Microbead Using a Miniaturized Silicon Hall Sensor. *Appl. Phys. Lett.* **2002**, *80* (22), 4199-4201.

(3) Di Michele, L.; Shelly, C.; Gallop, J.; Kazakova, O. Single Particle Detection: Phase Control in Submicron Hall Sensors. *J. Appl. Phys.* **2010**, *108* (10), 103918.

(4) Florescu, O.; Mattmann, M.; Boser, B. Fully Integrated Detection of Single Magnetic Beads in Complementary Metal-Oxide-Semiconductor. *J. Appl. Phys.* **2008**, *103* (4).
